# Osteoarthritis: New Insight on Its Pathophysiology

**DOI:** 10.3390/jcm11206013

**Published:** 2022-10-12

**Authors:** Stefano Coaccioli, Piercarlo Sarzi-Puttini, Panagiotis Zis, Giuseppe Rinonapoli, Giustino Varrassi

**Affiliations:** 1 European League Against Pain, 8000 Zurich, Switzerland; 2 Milano University, 20090 Milano, Italy; 3 Attikon University Hospital, National & Kapodistrian University, 157 72 Athens, Greece; 4 Medical School, University of Cyprus, Nicosia 1678, Cyprus; 5 Perugia University, 06126 Perugia, Italy; 6 Paolo Procacci Foundation, 00193 Rome, Italy

**Keywords:** osteoarthritis, physiopathology, subchondral bone, synovium, inflammation

## Abstract

Understanding of the basis of osteoarthritis (OA) has seen some interesting advancements in recent years. It has been observed that cartilage degeneration is preceded by subchondral bone lesions, suggesting a key role of this mechanism within the pathogenesis and progression of OA, as well as the formation of ectopic bone and osteophytes. Moreover, low-grade, chronic inflammation of the synovial lining has gained a central role in the definition of OA physiopathology, and central immunological mechanisms, innate but also adaptive, are now considered crucial in driving inflammation and tissue destruction. In addition, the role of neuroinflammation and central sensitization mechanisms as underlying causes of pain chronicity has been characterized. This has led to a renewed definition of OA, which is now intended as a complex multifactorial joint pathology caused by inflammatory and metabolic factors underlying joint damage. Since this evidence can directly affect the definition of the correct therapeutic approach to OA, an improved understanding of these pathophysiological mechanisms is fundamental. To this aim, this review provides an overview of the most updated evidence on OA pathogenesis; it presents the most recent insights on the pathophysiology of OA, describing the interplay between immunological and biochemical mechanisms proposed to drive inflammation and tissue destruction, as well as central sensitization mechanisms. Moreover, although the therapeutic implications consequent to the renewed definition of OA are beyond this review scope, some suggestions for intervention have been addressed.

## 1. Introduction

Osteoarthritis (OA) is the most common chronic articular disease with an increasing prevalence due to population aging and obesity [1,2,3]. Osteoarthritis is characterized by articular cartilage degeneration and persistent pain, causing disability, loss of function, decreased quality of life (QoL), and economic burden [4,5,6].

The global incidence of OA has been estimated at about 20% [7]. However, according to recent assessments, regional and individual country differences exist [7,8,9]. Indeed, OA incidence has been estimated at 10–17% in Europe, 12–21% in North America, 2–4% in South America, 16–29% in Asia, Africa, and Middle-Eastern countries [9,10]. Considering that OA susceptibility is strongly affected by genetic and environmental risk factors, the assessment of OA epidemiology in different populations can contribute to the understanding of the worldwide burden of the disease and may also shed light on the underlying mechanisms of the disease.

Osteoarthritis has long been considered a degenerative cartilage disease, characterized by a progressive loss of functionality due to different factors, such as excessive body weight, advanced age, surgical joint treatments, repeated joint injuries, and genetic predisposition [11,12]. Nevertheless, the understanding of the basis of OA has seen some interesting advancements in recent years [1,13,14]. Indeed, modern imaging approaches have shown that OA pathogenesis involves the breakdown of cartilage and structural changes in the whole joint [1,6,14]. In particular, it has been observed that cartilage degeneration is preceded by subchondral bone lesions, suggesting a key role of this mechanism within the pathogenesis and progression of OA, as well as the formation of ectopic bone and osteophytes [5,12,15,16,17].

Moreover, low-grade, chronic inflammation of the synovial lining now plays a central role in defining OA pathophysiology, and innate but adaptive central immunological mechanisms are now considered crucial in driving inflammation and tissue destruction. [3,14]. Lastly, the role of neuroinflammation and central sensitization mechanisms as underlying causes of pain chronicity has been characterized [18,19].

This evidence has led to a renewed definition of OA, which is now intended as a complex multifactorial joint pathology caused by inflammatory and metabolic factors underlying joint damage [1,3,6,14]. This new perspective directly impacts the definition of the correct therapeutic approach to OA. Therefore, an improved understanding of these pathophysiological mechanisms is crucial.

This narrative review provides an overview of the most updated evidence on OA pathogenesis. It presents the latest insight on OA pathophysiology, describing the interplay between immunological and biochemical mechanisms proposed to drive inflammation and tissue destruction.

## 2. Methods

A PubMed search was done using different combinations of pertinent keywords (e.g., “osteoarthritis” AND “pathophysiology”; “osteoarthritis” AND “synovium”, “osteoarthritis” AND “subchondral bone”) focusing on papers published in English over the past 5 years (2018–2022). If relevant, previously published papers were exceptionally considered, as judged by the Authors, as well as documents from the Authors’ collection of literature. Papers were selected for inclusion according to their relevance to the topic, as judged by the Authors.

### 2.1. Changes in the Osteochondral Unit during Osteoarthritis: The Role of the Subchondral Bone

Increasing evidence suggests that OA is a whole-joint disease in which all the joint components (cartilage, synovium, subchondral bone, and associated muscles) are affected [1,3,6] Articular cartilage covers the ends of bones in the diarthrodial joint and absorbs shock from joint movement. It has an aneural, avascular, and lymphatic structure composed of 65–80% water, 20–40% extracellular matrix (ECM), and 1–5% chondrocytes [20,21].

**Figure 1:**
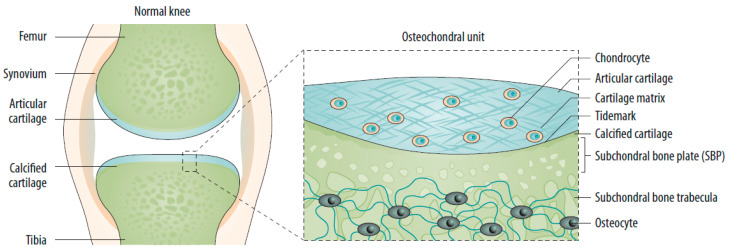
The normal joint—e.g., the knee—is composed of two articulating bones (femur and tibia), the articular cartilage, and the synovial lining of the joint cavity. A thin layer of calcified cartilage is present underneath the articular cartilage. The subchondral bone beneath the calcified cartilage is formed from cortical bone that merges into a network of trabecular bone, which is relatively porous and metabolically active. Source: original.

The subchondral bone is the bony layer below the hyaline cartilage and can be divided into the subchondral bone plate (SBP) and subchondral bone trabeculae (SBT). The SBP is a compact, porous, calcified plate traversed by nerve fibers and vessels. The SBTs are cancellous bony structures that undergo continuous bone remodeling [5,21]. Articular cartilage, calcified cartilage, SBP, and SBTs form the osteochondral unit, which transfers load during joint movement (Figure 1) [20,22]. Subchondral bone provides both nutritional and mechanical support for cartilage within the osteochondral unit, indicating that changes in the subchondral bone microenvironment can affect cartilage metabolism [5,17,20,22].

**Figure 2:**
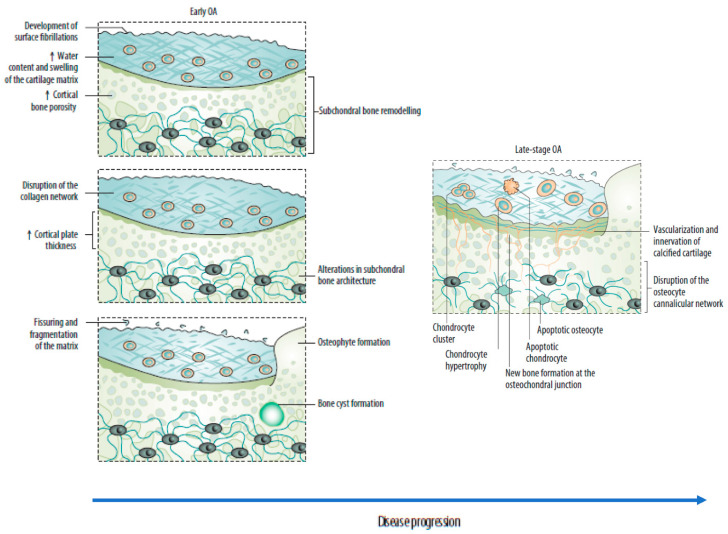
Sequential changes in the osteochondral unit during the evolution of osteoarthritis. (**Left**) Early OA is characterized by increased remodeling of the subchondral bone plate. With disease progression, loss of cartilage matrix proteoglycans and erosion of the collagen network led to the development of deep fissures and delamination of the cartilage, with exposure of the underlying zones of calcified cartilage and subchondral bone. In the subchondral bone, cortical plate thickness gradually increases. (**Right**) Chondrocytes exist mostly in clusters in late-stage OA, but chondrocyte apoptosis is also evident. In the deeper zones, chondrocytes undergo phenotypic alterations, developing features of a hypertrophic phenotype. The calcified cartilage expands and advances into the overlying hyaline articular cartilage, with duplication of the tidemark. This process is initiated by penetrating vascular elements, and accompanying sensory and sympathetic nerves, into the osteochondral junction. OA: osteoarthritis. Source: original.

Subchondral bone marrow lesions (SBMLs), reported by abnormal MRI signals below the calcified cartilage, have affected more than half of asymptomatic individuals over 50 years. Their prevalence seems to increase with age [5]. The SBMLs are due to abnormal and persistent mechanical insults, which lead to cellular and biomolecular responses to microfractures. Since SBMLs can be observed in the early stage of OA, and the worsening of SBMLs based on MRI manifestations has been associated with subsequent radiographic findings and persistent pain, they are thought to be helpful in early screening [5,17,23]. The site of an SBML is characterized by a high in situ turnover rate, pain, and activation of proinflammatory pathways, finally resulting in increased subchondral sclerosis and bone mineral density [5,18]. Clinical observations over 24 months suggested a strong relationship between the increased size of the SBMLs and cartilage volume loss in corresponding regions [5,17]. Consequently, subchondral bone remodeling is now considered a key element of OA, which can disrupt the integrity of the osteochondral unit and lead to increased crosstalk between cartilage and subchondral bone (Figure 2) [5,17,22]. A recent study reported the presence of a horizontal fissure at the interface within the osteochondral unit in obese patients, characterized by irregular cartilage erosion, fibro-granulation tissue infiltration, presence of free cartilage/bone debris, and rupture of micro capillaries [24]. This can be considered a new type of pathological feature, where neurovascular invasions have also been identified in degenerative osteochondral tissues (see following sections).

### 2.2. Risk Factors

The risk factors for OA can be divided into individual susceptibility features (increasing age, obesity, female sex, joint biomechanics, genetic factors) and factors that alter the biomechanical stability of joints (injury, repetitive joint use through occupation or leisure, and joint malalignment) [11,12,25].

#### 2.2.1. Individual Risk Factors

The increasing incidence of OA with age can be related to cumulative exposure to various risk factors and age-related biological changes in the joint structures [2,26]. Female sex and obesity have been strongly correlated with knee OA [27]. Knee malalignment and knee extensor muscle weakness have also been defined as moderate to strong risk factors [28].

Female sex and obesity are less represented risk factors for hip OA, but cam deformity or acetabular dysplasia have been found to moderately-to-strongly increase OA risk [6] Cam deformity and mild dysplasia have been found to increase the risk of OA, especially in middle-aged (55–65 years) patients; otherwise, a strong association between hip OA and severe dysplasia has been described, leading to its development at an early age (<50 years) [29].

Some studies supported the theory of diabetes as an independent risk factor for OA. One study, examining metabolic risk factors and their role in knee OA, showed a significant association between impaired glucose tolerance and knee OA after adjusting for age and biological sex, but not BMI [30,31,32]. In contrast, other more recent studies showed no association between diabetes and OA [33,34,35]. The differences in the results obtained across these studies can be attributed to other OA common risk factors, such as obesity and aging. However, these findings have not conclusively established the exact role diabetes plays in OA.

#### 2.2.2. Genetics

The contribution of genetics in OA is estimated to be between 40% and 80%, with a stronger genetic contribution in hand and hip OA than knee OA [25,36]. Rare mutations in monogenetic disorders associated with OA can result in early-onset OA. In contrast, late-onset OA is often characterized by a multifactorial clinical picture composed of common DNA variants and other risk factors. The effect size of these common variants is generally small [21,26]. Of note, among the 70 putative genes identified in genome-wide association studies in OA, no inflammatory genes can be detected; otherwise, growth factor clusters are strongly represented [37,38]. These include variants in *TGF-*β family genes, including ligands (TGFB1, GDF5), latent binding proteins (LTBP1, LTBP3), and signaling molecules (SMAD3). The FGF family is also represented. Collectively, these results underline the role of the loss of reparative features within the joint in the development of OA [39].

### 2.3. Joint-Related Factors

Heavy work activities are risk factors for both hip and knee OA; employment in farming or the construction industry is especially associated with hip OA, and work that involves frequent kneeling and heavy lifting is associated with knee OA [27].

Increased risk of OA has been found among athletes active in different sports [40]. Several high-impact sports (e.g., football, handball, hockey, wrestling, weight-lifting, and long-distance running) have been reported as moderately to strongly associated with an increased risk of hip or knee OA, often with a dose-response dependency [41,42]. For knee OA, the increased risk with sport is partly because of knee injuries; for hip OA, the risk might be associated with cam impingement, which can develop during sporting activities in adolescents [6]. Otherwise, in the first study examining the association between objectively measured physical activity and risk of developing knee OA in a community-based cohort of middle-aged and older adults at high risk for symptomatic knee OA, moderate–vigorous physical activity was not associated with incident knee OA [43].

Lastly, an association between the increasing use of technology, computers, and smartphones and hand OA has yet to be proved but is a common concern that requires further investigation, considering the increasing prevalence of these technologies in our lives [4].

## 3. Mechanisms Underlying Joint Deterioration

### 3.1. The Interplay between Immunological and Biochemical Processes

Increasing evidence highlights the interplay between mechanical damage to the osteochondral unit and low-grade chronic inflammation of the synovial membrane (synovitis) in the OA physiopathology. The involvement of innate and adaptive immune responses in the initiation and maintenance of inflammation is also described (Figure 2) [3,14].

### 3.2. Synovitis

Most OA patients present with low-grade inflammation, which has thus been crucial in OA development and progression [13,44]. Pro-inflammatory mediators, such as cytokines, lipid mediators, and reactive oxygen species (ROS), which are also produced by chondrocytes, synoviocytes, and osteoblasts, are responsible for battering anabolism and release of proteolytic enzymes, degrading the extracellular matrix and mediating cartilage loss [45]. Thus, an altered balance between pro- and anti-inflammatory cytokines, directed toward catabolism, can be described [1] Several pro-inflammatory cytokines, such as interleukin (IL)-1β, IL-6, IL-15, IL-17, IL-18, tumor necrosis factor (TNF)-α, and leukemia inhibitory factor (LIF), increased in OA tissues; at the same time, interferon-γ (IFN-γ), IL-6, IL-10, IL-4, and TGF-β provided anti-inflammatory activity [3].

Reactive oxygen species and inflammation are interdependent, each being the target of the other [46]. Reactive oxygen species production and oxidative stress were found elevated in patients with OA, and the concept of chronic or prolonged ROS production is considered central to the progression of inflammatory disease [45,47]. Evidence for ROS implication in cartilage degradation derives from lipid peroxidation products, such as oxidized low-density lipoprotein, nitrite (NO^2−^), nitrotyrosine, and nitrated (NO^3−^) products in the biological fluids and the cartilage of OA patients with and in OA animal models [47,48]. Otherwise, antioxidant enzymes, such as superoxide dismutase (SOD), catalase (CAT), and glutathione peroxidase (GPX) were decreased in OA patients, further suggesting a role for the oxidative stress in OA pathogenesis [45].

### 3.3. Innate Immune System

It has been observed that damage to cellular and cartilage ECM can generate damage-associated molecular patterns that activate the innate immune system and elicit a sterile inflammatory response through interaction with particle recognition receptors, such as Toll-like receptors (TLR), on the surface of immune cells, or with particle recognition receptors in the cell cytoplasm, such as nod-like receptors [44,49].

TLR-2 and TLR-4 have been found to be upregulated in the synovial tissue, articular cartilage lesions, and the synovial membranes of patients with OA, leading to the upregulation of matrix metalloproteases, nitric oxide, and prostaglandin E2 [44,50]. Recent reports suggest that among the TLR-induced innate immune responses, apoptosis is one of the critical events. Apoptosis is particularly important, given that chondrocyte death is a dominant feature in OA [44]. Once initiated, this inflammatory response leads to upregulation of catabolic factors, such as proinflammatory cytokines, proteolytic enzymes, and chemokines, and downregulation of anabolic factors, such as anti-inflammatory cytokines and growth factors, contributing to an ongoing sterile wound- healing “vicious circle” resulting in joint tissue pathology (Figure 1) [3].

The mediators that cause cartilage damage in OA, such as damage-associated molecular patterns and ECM components, can exude into the synovial fluid and activate synovial macrophages. Activated synovial macrophages further stimulate the release of proinflammatory cytokines, such as IL-1β and TNF-α and other catabolic as well as anabolic mediators involved in OA pathology [51].

Since macrophages are critical mediators for the maintenance of tissue homeostasis, they are thought to be involved in the pathology and symptomology of OA once dysregulated. Several studies have been published that further support macrophages as key mediators in OA-associated inflammation in recent years. Furthermore, it has been shown that modulating these cells or intervening with factors that modify their phenotypic state may be a promising approach to slowing down OA development [52,53,54,55,56]. Lastly, different recent studies suggested the involvement of mast cells in OA pathology due to the reported presence of these cells and their degranulation products in OA synovium and synovial fluid [57,58,59,60]. In particular, a central role for IgE-dependent mast cell activation has been proposed in the pathogenesis of osteoarthritis [58]. Moreover, the high prevalence of mast cells in the synovial fluid has been associated with structural damage in OA patients, further suggesting the role of these cells in the disease [60].

### 3.4. Adaptative Immunity

Macrophages can release pro-inflammatory cytokines that increase vascular permeability and further facilitate CD4+ T-cell infiltration, angiogenesis, and elevated levels of COX-2 in the OA synovium. CD4+ T cells and macrophages are abundantly present in OA synovium and can activate each other [61]. In particular, Th1 type T cells can initiate a cascade of events that activate both innate and adaptive immune responses, propagate synovial inflammation, and increase cartilage deterioration. T cells are also responsible for activating B cells, which hamper cartilage integrity by increasing inflammation and producing autoantibodies specific to chondrocytes’ surface proteins, such as collagen and osteopontin [62].

### 3.5. Neuroinflammatory Processes

Bidirectional interactions between the immune and nervous systems are increasingly understood to play a pathogenic role in chronic OA pain [18,19]. Communication between the two systems can occur at different levels: in the affected synovium, where nociceptors and macrophages interact, in the dorsal root ganglion, which can become infiltrated by macrophages in response to peripheral inflammation, and in the spinal cord dorsal horn, where microglia can modify synapses between nociceptors and second-order neurons [19].

People with OA are more sensitive to experimental noxious stimuli at body sites distant from their affected joints than unaffected people, suggesting the presence of central sensitization [63]. Since it is unrelated to radiological findings, this suggests that its role in osteoarticular pain is crucial [64,65]. Mast cells appear to be directly involved in neuropathic pain mechanisms, acting as interlocutors of microglia and promoters of central sensitization [18]. Direct nerve fiber damage can induce mast cell degranulation by releasing neuropeptides. Massively releasing mast cell mediators, such as histamine and NGF, enhance and support electrophysiological alterations of nerve fibers, leading to their sensitization [66,67]. Persistent sensitization of peripheral neurons is the first phase of central sensitization. In fact, neuronal hyperexcitability is followed by excessive release of neurotransmitters from peripheral terminals and those that connect to the dorsal horn of the spinal cord. This leads to the hyperexcitability of second-order neurons and activation of microglia [18].

### 3.6. Neoangiogenesis

An early diagnostic feature of OA is represented by the increased subchondral bone angiogenesis and the blood vessel invasion into the avascular cartilage [5,6]. Angiogenesis has proven to be a key factor in the pathogenesis of OA, facilitating the invasion of inflammatory cells and increasing local pain receptors that contribute to structural damage and pain [68].

Although antiangiogenic and angiogenic factors can be upregulated in the OA joint, the articular cartilage loses its resistance to vascularization, and vascular growth predominates. Angiogenesis in this location is closely related to the creation of channels into noncalcified articular cartilage from subchondral bone spaces and sensory nerve growth through shared regulatory pathways [68]. This involves releasing proangiogenic factors that stimulate nerve growth and molecules produced by vascular cells that stimulate and guide nerve growth [1]. Sensory nerves grow along new blood vessels in OA joints and invade noncalcified articular cartilage, osteophytes, and the inner regions of menisci. These structures are normally not innervated; thus, nerve and blood vessel invasion contribute to pain [5].

### 3.7. Osteophyte Formation and Joint Remodeling

A characteristic feature of OA is the formation of osteophytes, which are osteocartilaginous outgrowths that typically form at the joint margins [69]. Osteophytes are usually considered an endogenous repair response to excessive mechanical load in degenerative joints, attempting to stabilize the involved joint to better bear the abnormal force [15,70]. In recent studies, osteophyte formation has been reported to be associated with abnormal mechanical forces that result in lateral thrust, joint instability conditions, and malposition of the meniscus; therefore, control of abnormal joint movement and instability may be a beneficial precautionary measure for OA progression [71,72]. It has also been proposed that osteophyte formation may play a compensatory role in redistributing forces to protect articular cartilage [73]. For instance, a link between the immunological mechanisms related to OA and osteophyte pathology has been proposed by a recent work, which provided evidence for mast cell presence in osteophytes of OA joints and demonstrated their maturation mediated by the synovial fluid synchronously with disease progression [57].

### 3.8. Functional Outcomes of Osteoarthritis

It has been estimated that OA causes limitations in activity in 7% of the older adult population; 25% cannot perform major activities of daily living, and about 80% of OA patients present some movement limitation [74]. Other than activity limitations, the individual burden of OA includes pain and markedly reduced QoL. Since OA is a chronic disease, the accompanying chronic pain can be intermittent but generally severe or intense, or persistent [75].

### 3.9. Small Joints

Wrist OA usually results from a post-traumatic sequel and selectively arises in the joints that involve the scaphoid bone. Although well tolerated for many years, it can result in severe functional impairments consequent to mechanical pain, motion range limitation, and decreased strength [76]. These symptoms develop gradually in most patients but may be precipitated by an injury or unusual sustained activity [77]. In addition to motion range limitation, the physical findings may include swelling, usually at the dorsal-radial aspect of the wrist, related to a combination of osteophytes and focal synovitis [78].

Osteoarthritis of the fingers is an especially common condition in postmenopausal women. Finger OA destroys interphalangeal cartilage and results in pain, swelling, decreased finger motion, joint deformities, and difficulty performing activities that require grip or pinch [79].

### 3.10. Middle and Large Joints

Middle and large joint OA is mainly characterized by pain and inactivity [80]. Much of the focus on disability is on the knee because of its high prevalence (83% of the total OA burden) and attendant disability. It has been estimated that 11% of adults with knee OA need personal care assistance, and 14% require help with routine activities [74]. Notably, hip and knee OA have been related to a ~20% excess mortality as compared with age-matched controls, due in part to lower levels of physical activity [81].

## 4. Discussion

Unraveling the disease processes in OA has always been challenging because of its nature, characterized by tissue inaccessibility and heterogeneity of clinical phenotype [82,83]. Different pathophysiological mechanisms involved in OA development and progression have been described in recent years [1,13,14]. It is now widely accepted that OA pathogenesis involves the breakdown of cartilage and the remodeling of subchondral bone, formation of ectopic bone and osteophytes, hypertrophy of the joint capsule, and inflammation of the synovial lining. Consequently, it is now recognized that low-grade, chronic inflammation has a central role in the pathogenesis of arthrosis. In addition, the chronicity of pain in people with OA is increasingly recognized to be related to central sensitization mechanisms [18,19]. Although the therapeutic implication consequent to the renewed definition of OA is beyond the scope of this review, this more complex clinical picture has inevitable therapeutic consequences and provides numerous suggestions for intervention, acting on the various pathogenetic processes. Firstly, this allows a better understanding and identification of different patient phenotypes and the detection of the disease in its early stages, as well as the possibility to identify patients who are at higher risk of progression, which in turn could be used to guide clinical decision-making and develop more effective and specific therapeutic interventions [12]. In addition, integration of the central sensitization concept during clinical reasoning and patient management should also be included.

There are aspects still poorly explored, e.g., influence of ethnicity and lifestyle. This could potentially explain the epidemiological differences existing between countries such as Italy [84], and the USA [85].

A further, and important aspect would be a more profound study of the influence of comorbidities, and other interesting players, such as oxidative stress [86]. More precise and organic studies on the interactions between anabolic/catabolic processes, diabetes, and degeneration of the cartilages would be much more useful than the sectorial or sporadic existing ones [87,88,89].

According to the new evidence, the prevention and the conservative treatment of OA acquire an important role in a surgical approach. Consequently, interventions must be emphasized to counteract the modifiable variables that drive disease progression. These interventions should include, for example, treatments for obesity and approaches for improving joint mechanics. Importantly, these interventions must be instituted before considerable structural and functional alterations develop in the joint tissues. At the same time, chondroprotection can be reconsidered as a first-line approach to OA, particularly in the early stages when the cartilage tissue is sensitive to protective and structural reconstruction interventions to avoid more invasive therapeutic interventions.

People suffering from lower-extremity OA should be encouraged to perform physical activity, even if of modest intensity [90]. There was no evidence to suggest accelerated OA progression for physical activity below 10,000 steps per day [91]. Otherwise, it has been suggested that physical activity can decrease pain and improve physical function and health related QoL in hip or knee OA cases [91]. Moreover, exercise has been found to reduce evidence of central sensitization in many patients with chronic pain conditions [92].

## 5. Conclusions

OA is now intended as a multifactorial pathology caused by different biological functional alterations based on joint damage. A close understanding of the active biological and cellular processes that contribute to disease pathology at a given stage of OA progression is fundamental to targeting the individual components of the pathophysiological process with specific therapeutic agents and detecting them at the earliest stages. In this perspective, the prevention and the conservative treatment of OA acquire an important role.

## Data Availability

Not applicable.
